# Ipsilateral Testicular Catch-Up Growth Rate Following Microsurgical Inguinal Adolescent Varicocelectomy

**DOI:** 10.1100/2012/356374

**Published:** 2012-07-31

**Authors:** Orhun Sinanoglu, Seyit Erkan Eyyupoglu, Sinan Ekici

**Affiliations:** ^1^Department of Urology, Maltepe University School of Medicine, Maltepe, 34843 Istanbul, Turkey; ^2^Urology Clinic, Amasya State Hospital, 05100 Amasya, Turkey

## Abstract

*Objective*. To evaluate the ipsilateral catch-up growth rates compared to contralateral testicular growth in adolescents with varicocele undergoing microsurgical inguinal varicocelectomy. *Materials and Methods*. Between December 2005 and May 2007, 39 adolescent patients with grade 2-3 varicocele admitted to our clinic with complaints of pain and/or testicular asymmetry were operated. Preoperative mean age was 14.5 ± 1.96 (9–17). Testicular volumes were assessed with ultrasound every 3 months. The available followup was 39 months. *Results*. In our series, mean testicular preoperative volumes were 9.07 ± 3.19 mL for the right and 5.90 ± 1.74 mL for the left. Mean testicular volumes at the end of follow up were 13.97 ± 3.42 mL for the right and 12.20 ± 4.05 mL for the left. The testicular catch-up growth approximately begins after the 9th month and significant catch-up occurred in the 12–24 months (*P* < 0.05). *Conclusion*. Since testicular volume is the primary method of assessing testicular function in adolescents, testicular size can predict future fertility status significantly 9 months after surgical varicocele correction.

## 1. Introduction

The varicocele is the most common surgically reversible cause of male infertility. The prevalence of varicocele is approximately 15–20% in general population and 30–40% among infertile men [1]. There is a correlation between testicular hypotrophy and varicocele in adolescent and adult patients [2]. Idiopathic varicocele is a frequently diagnosed andrological disease in adolescent age and the most common treatable cause of male infertility [3]. In several studies, the negative effect of adolescent varicocele on testicular volume and the relationship between varicocele and the decline in testicular growth have been described [4–6]. 

It is now accepted that the treatment of varicocele during childhood should be concomitant with the onset of ipsilateral testicular growth arrest (or testicular hypotrophy) [6, 7]. Although changes in semen quality are considered as the first indication for surgery in adults, it is usually accepted that the treatment of varicocele in adolescence is indicated with the onset of testicular growth retardation and testicular pain, considering that these patients may manifest impaired fertility in adult age [7]. A 60–89% catch-up growth rate of the affected testis in adolescent patients after surgical repair has been well documented, since many studies reported early reversal of testicular hypotrophy after varicocelectomy [8–11]. In order to evaluate testicular improvement after surgery, the term “catch up growth” was introduced first by Kass and Belman into the literature [12]. This compensatory increase in testicular size which was found to be correlated with functional improvement [8, 10]. Both clinical and ultrasonographic examinations are performed to confirm ipsilateral catch-up growth and to rule out recurrence as well [12].

The aim of the study is to evaluate the ipsilateral testicular growth improvement in the follow up period after varicocele surgery in adolescents and to assess the interval where the significant testicular catch-up growth rate occurs compared to contralateral one.

## 2. Materials and Methods

Between December 2004 and May 2007, 39 adolescent patients having testicular asymmetry with or without pain in standing and lying position with varicocele on the left testis were enrolled in the study. Their ages ranged from 12 to 17 years (mean ± SD, 14.5 ± 1.96). Patients were followed with history and physical and ultrasound measurements. Varicoceles were graded by either of two attending urologists. A grade 1 varicocele was palpable during a Valsalva maneuver, grade 2 was palpable without a Valsalva maneuver, grade 3 was visible on inspection of the scrotum. Thirty-nine operated patients had venous diameters >2.7 mm (mean = 3.4 ± 0.6 mm) [13].

The inclusion criteria for this study were patient age of 9–18 years, left varicocele, testicular hypotrophy at ultrasound, and complete follow-up data every three months (minimum 39 months duration) after surgery. Bilateral varicocele, recurrenceand persistence of varicocele, testicular atrophy after surgery, hydrocele any scrotal pathology other than varicocele, history of varicocelectomy, previous testicular trauma or previous inguinal and scrotal surgery, neurologic or metabolic diseases and previous infections of the urinary system were exclusion criteria. Informed consent was obtained from all patients, and the study protocol was approved by the ethics committee of our institution. Preoperative testis volumes were calculated in mL with its anteroposterior × cephalocaudal × transversal lengths × 0.523 by US [14].

Mean preoperative ipsilateral testis volume was 5.90 mL ± 1.74 and that of the contralateral was 9.07 mL ± 3.19. All patients underwent inguinal microscopic artery sparing varicocelectomy under general anesthesia. Patients were called every three months for US control, hormonal profiles, and semen analyses were obtained when possible. The unilateral testicular growth rate was calculated with the difference of testicular size within the interval of two control visits and presented as percentage values. The percentage of growth rate difference between preoperative and postoperative testicular volumes during followup for both testes were calculated to establish the time point where the ipsilateral growth became significantly increased compared to contralateral one. The results were analyzed using analysis of variance for repeated measurements (ANOVA) with the Statistical Package for Social Sciences, version 16 for Windows. Significance value was set at *P* < 0.05.

## 3. Results

The prospective observation of our varicocele patients revealed that 12% of them were adolescent. Operated adolescent varicoceles were bilateral in a rate of 3/42 (7.14%). Of the 39 operated unilateral varicocele patient, 31 were included in this study since they had testicular hypotrophy (79%). Among these, one patient had recurrence verified by US and one had hydrocele that required operation. Additionally, three patients were lost to followup. Excluding these, 26 patients with mean age of 14.5 ± 1.96 had remarkable improvement in their testis. Mean preoperative ipsilateral testis volume was 5.90 mL ± 1.74 and that of the contralateral was 9.07 mL ± 3.19. The mean age at the end of the followup in May 2009 was 17 ± 1.97 (Table 1). Mean ipsilateral and contralateral testicular volumes were grown from 5.90 to 12.65 mL and from 9.07 to 13.97 mL, respectively, at 39 months postoperatively (Table 1).

**Figure 1:**
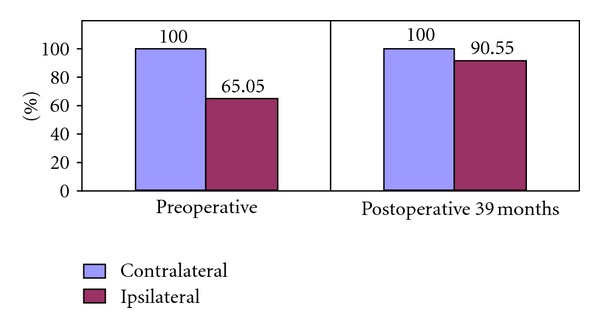
The effect of varicocelectomy on ipsilateral testis development (mean contralateral testis volume was admitted as 100 units).

**Figure 2:**
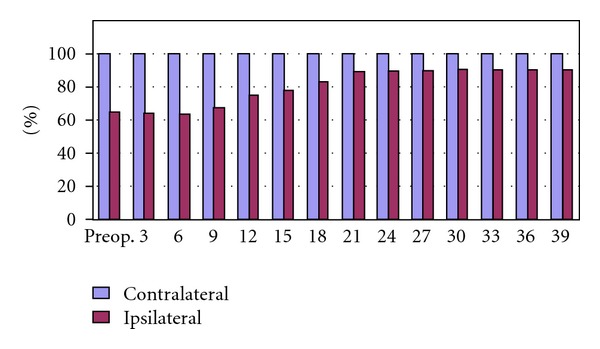
Bilateral testis volumes in control intervals (mean contralateral testicular volume was admitted as 100 units).

**Figure 3:**
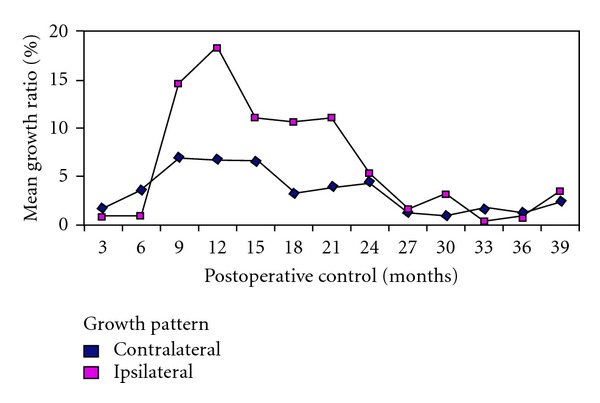
Percentage of growth change in ipsilateral and contralateral testes during control periods.

Ipsilateral testis/contralateral testis ratio was 65.55% preoperatively and this increased to 90.55% at the last followup (Figure 1). The preoperative values and those at the end of the followup are shown in Table 1. The dynamics of this testicular volume changes in control intervals were also evaluated in which mean contralateral testicular volume was admitted as 100 units. Significant postoperative ipsilateral growth pattern was obtained in 12–39 months of followup. Ipsilateral testicular growth catch up rates began to increase after the 9th month, and became significant between 12–24 months (Figures 2 and 3) (*P* < 0.05).

## 4. Discussion

The evidence suggest that varicocelectomy is beneficial to adolescents with ipsilateral testicular hypotrophy since untreated varicocele was shown to have progressive deleterious effects on the future fertility status [15]. Even in the earliest description of varicocele, testicular volume decrease was taken into consideration. An earlier study reported testicular atrophy incidence as 74% [16]. The ipsilateral testicular volume catch-up growth rate or hypertrophy has been one of the most important endpoints predicting the outcomes of varicocelectomy. Since it is not usually possible to perform preoperative semen analysis in pediatric age group and established clinical criteria to properly define the postoperative outcomes are absent, the improvement in the testicular volume has been considered to be the main outcome measure [12].

In an early study with 72 patients with preoperative left testicular volume loss of 2 cc or greater comparing 36 patients undergoing artery sparing varicocele operation to 36 patients following Palomo procedure in a mean follow-up of 22 months, authors found out that mean preoperative relative left testicular volume was increased from 73 to 91% in artery sparing arm compared to the increase from 72 to 92% in Palomo arm. The relative volume increase was 3% in control groups [10]. Two operative techniques were compared according to their success rates, testicular growths in both groups were statistically significant but no difference was present between two procedures [10]. Twenty-seven patients having preoperative small testis and postoperative testicular catch-up were followed in another series and the initial left to right testicular ratio of 67% was found to be increased to 94% postoperatively. The average follow-up periods were given as 3.7 years [17].

In a larger recent series, the authors evaluated varicocelectomies in 465 patients aged 9–14 years and compared the laparoscopic artery-preserving varicocelectomy and open inguinal microscopic artery-preserving varicocelectomy with a venous-venous bypass. They measured testicular volume before and after surgery using US. Although the overall catch-up growth rate for both groups was found to be 80%, after 18 months, only 45% of patients in laparoscopic and 34% of patients in inguinal varicocelectomy group had equal bilateral testicular volume. None of these surgical procedures had a statistically significant correlation with age at surgery, varicocele size, or catch-up rate. The semen analysis results did not show statistically significant differences either between the two groups [12].

As to our study, twenty-six of 31 patients (83.87%) having ipsilateral hypotrophy had testicular growth. In these patients (*n* = 26) initial relative ipsilateral to contralateral testicular ratio was 65.05%. A mean relative volume of 90.55% was obtained at postoperative 39th month which seemed to be in accordance with the authors suggesting that ipsilateral testicular hypertrophy occurs in a substantial number of adolescents following varicocele ligation [18]. However, the significance in postoperative increase in size of the testis has been denied in a very recent study which also suggested the significant role of elevated FSH in detecting suboptimal outcomes following varicocele surgery [19]. The ipsilateral testicular hypertrophy is attributed to destruction of lymphatics during surgical procedure not to varicocele correction [20]. This hypertrophy occurred mostly after nonlymphatic sparing varicocelectomies. Since the lymphatics of testis in our study were spared under microscopic varicose vein ligation, the ipsilateral testicular growth was not thought to be due to the lymphatics harm in accordance with the reports suggesting that surgical modality sparing the testicular lymphatics revealed better catch-up growth rates [19, 20].

Indeed, there are several limitations of our study; first, the sample size was small due to poor response rate of patients in that age group during followup; second, since the semen analysis and FSH levels were obtained only in a very small number of patients, they could not be included in the study. And lastly, testicular histomorphological examination was not possible due to ethical considerations.

In conclusion, the compensation of testicular size discrepancy in adolescent with varicocele that complained about testicular asymmetry remains a important issue. Although the catch-up growth phenomenon after adolescent varicocelectomies was reported in earlier and recent studies, a follow-up itinerary remains to be determined. In our study we observed that ipsilateral testicular catch-up growth rate began at 9-month postoperatively and continued until the 36th month. Further studies with larger samples enrolling matched age groups may lead to new nomograms in order to evaluate the postoperative outcomes in adolescent patients with varicocele.

## Figures and Tables

**Table 1:** The summary of preoperative and postoperative data of adolescent varicocele patients.

Data (#26)	Preoperative	Postoperative 39 months
Ipsilateral testicular volume	5.90 ± 1.74	12.65 ± 4.05
Contralateral testicular volume	9.07 ± 3.19	13.97 ± 3.42
Bilateral testicular volume difference (mL)	3.16 ± 2.27	1.38 ± 1.59
Ipsilateral/contralateral testis volume ratio (%)	65.05 ± 16.40	90.55 ± 12.60
Age	14.5 ± 1.96	17 ± 1.97

## References

[1] Jarow JP (2001). Effects of varicocele on male fertility. *Human Reproduction Update*.

[2] Pasqualotto FF, Lucon AM, De Góes PM (2005). Testicular growth, sperm concentration, percent motility, and pregnancy outcome after varicocelectomy based on testicular histology. *Fertility and Sterility*.

[3] Lund L, Tang YC, Lee KH, Liu K, Yeung CK, Roebuck D (1999). Testicular catch-up growth after varicocele correction in adolescents. *Pediatric Surgery International*.

[4] Paduch DA, Niedzielski J (1997). Repair versus observation in adolescent varicocele: a prospective study. *Journal of Urology*.

[5] Laven JSE, Haans LCF, Mali WPTM, Te Velde ER, Wensing CJG, Eimers JM (1992). Effects of varicocele treatment in adolescents: a randomized study. *Fertility and Sterility*.

[6] Çayan S, Akbay E, Bozlu M (2002). The effect of varicocele repair on testicular volume in children and adolescents with varicocele. *Journal of Urology*.

[7] Zampieri N, Cervellione RM (2008). Varicocele in adolescents: a 6-year longitudinal and followup observational study. *Journal of Urology*.

[8] Sakamoto H, Ogawa Y, Yoshida H (2008). Relationship between testicular volume and varicocele in patients with infertility. *Urology*.

[9] Sakamoto H, Yajima T, Nagata M, Okumura T, Suzuki K, Ogawa Y (2008). Relationship between testicular size by ultrasonography and testicular function: measurement of testicular length, width, and depth in patients with infertility. *International Journal of Urology*.

[10] Atassi O, Kass EJ, Steinert BW (1995). Testicular growth after successful varicocele correction in adolescents: comparison of artery sparing techniques with the Palomo procedure. *Journal of Urology*.

[11] Kirk J, Pinto R, Kroovand L, Jarrow J (1994). Varicocele related testicular atrophy and its predictive effect upon fertility. *Journal of Urology*.

[12] Kass EJ, Belman AB (1987). Reversal of testicular growth failure by variocele ligation. *Journal of Urology*.

[13] Eskew LA, Watson NE, Wolfman N, Bechtold R, Scharling E, Jarow JP (1993). Ultrasonographic diagnosis of varicoceles. *Fertility and Sterility*.

[14] Krone KD, Carroll BA (1985). Scrotal ultrasound. *Radiologic Clinics of North America*.

[15] Preston MA, Carnat T, Flood T, Gaboury I, Leonard MP (2008). Conservative management of adolescent varicoceles: a retrospective review. *Urology*.

[16] Pozza D, D’Ottavio G, Masci P, Coia L, Zappavigna D (1983). Left varicocele at puberty. *Urology*.

[17] Parrott TS, Hewatt L, Belman AB (1994). Ligation of the testicular artery and vein in adolescent varicocele. *Journal of Urology*.

[18] Gershbein AB, Horowitz M, Glassberg KI (1999). The adolescent varicocele I: left testicular hypertrophy following varicocelectomy. *Journal of Urology*.

[19] Deshpande A, Cohen R, Tsang I, Ambler G, Fleming S (2011). The validity of testicular catch-up growth and serum FSH levels in the long-term postoperative assessment of laparoscopic varicocele correction in adolescents. *Urology Annals*.

[20] Kočvara R, Doležal J, Hampl R (2003). Division of lymphatic vessels at varicocelectomy leads to testicular oedema and decline in testicular function according to the LH-RH analogue stimulation test. *European Urology*.

